# How Donors Can Collaborate to Improve Reach, Quality, and Impact in Social and Behavior Change for Health

**DOI:** 10.9745/GHSP-D-21-00007

**Published:** 2021-06-30

**Authors:** Catherine Harbour, Hope Hempstone, Angela Brasington, Sohail Agha

**Affiliations:** a Vym Consulting.; b Formerly of U.S. Agency for International Development.; c U.S. Agency for International Development, Washington, DC, USA.; d Bill & Melinda Gates Foundation, Seattle, WA, USA.

## Abstract

To enable greater reach, quality, and impact of investments in social and behavior change, donors need to be intentional in building collaborative relationships that apply proven practices. We offer recommendations for maximizing the impact of donors' investments.

## Key Messages

- Since 2018, collaboration among funders of social and behavior change (SBC) in global health has markedly increased. Anecdotal evidence suggests that these efforts are beginning to reduce duplication of effort, improve support to implementers, and increase co-investment.
- As donor collaborations supporting SBC mature, it will be important that they move beyond information sharing to enable more integrated coordination, co-investment, and even cocreation of investments. Global and regional collaborations among donors must also seek to engage country governments and regional coordinating bodies as leaders in the work of SBC.
- To facilitate donor collaboration, our research suggests that participating donors should: (1) define group purpose, goals, and roles clearly and early on; (2) support host country leadership; (3) recognize and leverage the different strengths of private and public donors; (4) demonstrate commitment by investing resources; (5) use honest conversations about failure to inform a joint learning agenda; (6) encourage proactive communication and informal discussion; (7) take the time to understand collaborating organizations' grantmaking, procurement, and compliance processes; (8) consider using a trusted member (or an intermediary) to progress work; and (9) seek early wins that build confidence in the group.

## BACKGROUND

The development sector has long recognized the need for donors to collaborate effectively with host country governments, with each other, and with partners in civil society and the private sector. In 2005, the Paris Declaration sought to improve the quality of aid and its impact on development. Donor countries agreed to increase harmonization and coordination, simplify procedures, and avoid duplication by sharing information.^1^ By 2011, however, coordination remained a problem. The Working Party on Aid Effectiveness noted that^2^:

*Coordination of donors often remains weak precisely where working towards common goals is needed the most, and weak national leadership and capacity become an excuse for uncoordinated donor-driven approaches.*

The Sustainable Development Goals, set in 2015 by the United Nations General Assembly, include Goal #17, Partnerships for the Goals, and have encouraged better collaboration among stakeholders including donors.

This article uses the term “donor” to refer to both official development assistance (ODA) organizations and philanthropies. The Organisation for Economic Co-operation and Development (OECD) Development Assistance Committee defines ODA^3^ as:

*Government aid designed to promote the economic development and welfare of developing countries… Aid may be provided bilaterally, from donor to recipient, or channeled through a multilateral development agency such as the United Nations or the World Bank.*

According to the OECD, the total value of philanthropic funding for development is about 5% of the value of ODA, which was $US23.9 billion between 2013–2015.^4^ Private philanthropy invests more in the health sector, by far, than it invests in other development sectors. In 2018–2019, private philanthropy was the third-largest source of health-sector funding, after bilateral aid from the United States and World Bank Global Fund.^5^

### Benefits and Challenges of Donor Collaborations

Donor collaborations can support national governments to improve the scale and efficiency of their activities by reducing duplication of efforts and supporting local priorities more strategically. Research conducted on collaborations among donors in the United States found that a donor collaboration may increase the visibility of an issue or area of work. Donor collaboration can also facilitate access to nonfinancial, in-kind resources, including technical assistance, networks, consulting help, and convening of influence across donors.^6^ Finally, effective collaborations leverage the strengths of different donors, which supports the efficient function of the development ecosystem. A survey of donors in India found that the majority of respondents strongly agreed that working collectively enabled them to make greater progress on social challenges in India than working alone.^7^

Effective donor collaborations leverage the strengths of different donors.

Donor collaborations also have challenges. From an interpersonal and interorganizational standpoint, donor staff must be willing to compromise, relinquish a degree of control, and share credit for accomplishments in a collaboration. Collaborations require time and energy and may need to adapt as organizational members and staff change. Not surprisingly, interpersonal tensions can arise, for example, when members with access to greater financial resources and broader geographic scope overlook the unique perspectives of smaller, local members with more limited financial resources.^6^ From a logistical perspective, donor collaborations can be challenging to organize due to differing fiscal cycles and a lack of visibility into the fiscal processes of potential partner donor organizations.

## THE CASE FOR IMPROVED DONOR COLLABORATION IN SOCIAL AND BEHAVIOR CHANGE

Global health programming is grounded in mutually reinforcing investments in policy, supply chains, service delivery, and social and behavior change (SBC),* which uses evidence-based interventions to increase the adoption of healthy behaviors by individuals and influence the social norms that underpin those behaviors. SBC may be used to create demand for health products and services; promote the practice of healthy behaviors within the household and community; improve client-provider interactions; and influence community leaders and other decision makers.^1^

Recent years have seen a growing interest in SBC generally and demand creation specifically among donors, governments, and development implementing partners. This expanded interest, together with an increased appreciation of both the demand-side barriers to improved health and the limited funding available for SBC programming, research, and evaluation, has prompted donors to consider how best to align their investments.

Several parallel activities in 2017, including internal demand-side landscaping conducted by the Bill & Melinda Gates Foundation (BMGF) and consultations held by the United Nations Children's Fund (UNICEF) to inform the creation of a global mechanism for SBC, highlighted the need for improved donor collaboration and coordination on SBC in global health.^8^ In general, these exercises indicated that, while new donors and implementers were investing in SBC, many were operating in isolation and were unlikely to achieve population-level impact due to missed opportunities for learning and duplication of effort. These reflective exercises were followed shortly by the 2018 Global SBCC Summit, which emphasized the need for more coherent direction within the field of SBC. Inspired by these activities, representatives of major donors initiated a concerted effort to better align their respective investments through formal and informal activities.

New donors and implementers were investing in SBC, but many were operating in isolation and were unlikely to achieve population-level impact due to missed opportunities for learning and duplication of effort.

In December 2018, after discussions among program officers at BMGF, the U.S. Agency for International Development (USAID), and the Children's Investment Fund Foundation (CIFF), BMGF convened a meeting to discuss how demand and SBC could be better coordinated among donors and multilateral organizations. Staff from donor organizations, including the Agence Française de Développement, CIFF, the United Kingdom Foreign, Commonwealth, & Development Office (formerly United Kingdom Department for International Development), the European Commission, the William and Flora Hewlett Foundation, the David and Lucile Packard Foundation, Surgo Foundation, Unilever, USAID, the Wellcome Trust, the World Bank, UNICEF, and the World Health Organization, and FP2020 met to explore the potential for collaboration in their investments on SBC. Before this meeting, there had been few substantive donor collaborations focusing exclusively on SBC; more often, behavior change was addressed as a component of broader thematic or sectoral initiatives.

In preparation for this meeting, the meeting organizers—including the authors of this commentary—undertook program research, involving document review and in-depth interviews, to prepare a background paper for the meeting. We identified purposively the documents that we included in the review. As described in the next section, most of the literature we reviewed described models of donor collaboration and lessons learned from them based on collaborations among donors investing in a high-income country, the U.S., and none of them focused explicitly on donor collaboration in SBC.

To complement the perspectives on donor collaborations that we found in the literature, most of the semistructured in-depth interviews we conducted focused on experiences with donor collaborations related to SBC in low- and middle-income countries. Despite substantial differences in context, we found that the factors associated with success of donor collaborations in high-, middle-, and low-income countries were fairly consistent. We conducted semistructured interviews with donor staff, representatives of a host-country government, and researchers. These interviews were conducted with individuals in their professional capacities, either as staff of donor organizations or as staff of other stakeholders in the sector, such as implementing partners and research organizations. Ethics review was not deemed necessary. Between October 2018 and January 2019, we conducted a total of 26 interviews: 14 with donor staff who participated in the meeting, 2 with a host-country government, and 12 with other stakeholders familiar with donor coordination and/or with SBC investments. Interviewees were invited by email and implicitly gave their consent to participate by responding to the invitation email, scheduling the call, and participating in the interview. Interviews focused on participants' perspectives on donor collaboration. We then drafted a working paper to summarize our findings.

This commentary builds upon and expands that research. First, we present several models of donor collaboration, and offer examples of each from SBC investments. Then we identify factors associated with the success and failure of donor collaborations, particularly those focused on demand and behavior change in global health,^†^ to help define the way forward for donors and multilateral institutions exploring opportunities for collaboration in SBC.

We identify factors associated with the success and failure of donor collaborations to help define the way forward for donors exploring opportunities for collaboration in SBC.

## DONOR COLLABORATION MODELS

Research on donor collaborations in the US can help elucidate current and potential models for engagement among SBC funders investing in international development. Our research identified several models of donor collaboration that share some common features, including a recognition that collaboration occurs along a spectrum, from informal groups that are loosely structured, to more formal groups that are more structured and closely integrated. Three useful and similar models are described by GrantCraft, Catalyst of San Diego and Imperial Counties (formerly San Diego Grantmakers), and Bridgespan (Table). A fourth model, Collective Impact, developed by FSG, uses a specific and more highly structured approach.

**TABLE.** Typologies of Donor Collaborations, With Examples

Model	Less integrated ------------------ More integrated				
**Grantcraft**	Learning Network	Strategic Alignment Network	Pooled Fund		
**San Diego Grantmakers**	Learn	Plan	Act		
**Bridgespan**	Exchange	Coordinate	Co-invest	Create	Fund the funder
**Examples**	Global SBC Donor group Scaling Up Nutrition The Curve Community of Practice	Nigeria SBC Donor Coordination Committee, and similar groups in other countries Global Partnership to End Violence Against Children	Social Norms Learning Collaborative Adolescents 360 Generation Unlimited United Nations Trust Fund to End Violence Against Women U-Report	Bernard van Leer Foundation and Conrad N. Hilton Foundation co-funding INSEAD course on behavioral science for early childhood development	United Nations Children's Fund (UNICEF) Innovation Fund

Abbreviation: SBC, social and behavior change.

GrantCraft distinguishes 3 types of funder collaborations based on how the participating funders structure their work together. A learning network, the most loosely structured, is a group of funders that come together to share information, learn about developments in a field or issue area, and discuss potential ways to invest more effectively.^6^ A strategic alignment network, which is more structured, comprises funders who share a mission, develop strategies together, and work toward joint impact but do their grantmaking separately. By contrast, a pooled fund, which is the most structured, is a “pot” of money to which funders contribute and from which grants or program-related investments are disbursed. Money from the pot is often used without distinguishing its original donor. Similar in many ways to how a foundation functions, a pooled fund has staff to develop strategies, issue calls for proposals, and assess and select potential grantees.

Similar to the GrantCraft model is the “Learn-Plan-Act” model proposed by San Diego Grantmakers, which describes funders' motivations to work with other funders along a continuum of how committed they are to learning together, planning together, or acting together.^9^

Bridgespan expands upon the 3 types of collaboration identified by GrantCraft and San Diego Grantmakers, identifying 5 models of donor collaboration.^10^ The least integrated collaborations serve the purpose of exchanging ideas and raising awareness. The most integrated collaborations are re-granting organizations, in which more than 1 funder invests in another funder with expertise in a content area.

We reviewed a fourth model of donor collaboration, called Collective Impact (CI). Since 2011, FSG and other organizations have used the CI approach to collaborative problem solving and a structured, cross-sector approach to solving complex social problems with partners, including donors.^11^^–^^13^ Five essential conditions of a CI initiative are backbone support, a common agenda, mutually reinforcing activities, continuous communication, and shared measurement.^14^ An example of the CI approach in SBC is the advocacy work of Alive & Thrive, which improved infant and young child feeding policies in seven countries of Southeast Asia.^15^

## RECOMMENDATIONS FOR SUCCESSFUL DONOR COLLABORATION

Our research suggested the following practices support a successful donor collaboration: (1) define group purpose, goals, and roles clearly and early on; (2) support host-country leadership; (3) recognize and leverage the different strengths of private and public donors; (4) demonstrate commitment by investing resources: time, money, networks, and institutional clout; (5) use honest conversations about failure to inform a joint learning agenda; (6) encourage proactive communication and informal discussion; (7) take the time to understand collaborating organizations' grantmaking, procurement, and compliance processes; (8) consider using a trusted member (or an intermediary) to progress work; and (9) early wins build confidence in the group.

Examples related to SBC are cited in the text and Table, with more information in a Supplement.

### Define Group Purpose, Goals, and Roles Clearly and Early On

Effective collaboration requires a shared understanding of the problem to be solved, which may pertain to health outcomes, tactical and operational barriers to achieving those outcomes, or some combination thereof. Facilitators must also work to understand the politics, backstories, and relationships among group members that may impact the achievement of shared goals. Agreeing on some shared indicators of progress and success can help donors confirm that their purposes and goals are aligned.

Agreeing on some shared indicators of progress and success can help donors confirm that their purposes and goals are aligned.

For example, in Nigeria, an SBC committee was established in 2019 under the Donor Partners Group for Health (DPG-H). The SBC committee is guided by terms of reference that define shared objectives, scope, and modalities for effective collaboration among donors funding demand-side activities. The group's vision is a country with effective geographic and technical coordination of SBC investments that are aligned with Government of Nigeria priorities and that maximize opportunities for synergies, co-investment, and program impact.

### Support Host Country Leadership

Sustainable development requires the leadership of host-country governments. This principle is particularly true of SBC, given a historical over-reliance on donor funding in many countries and potential sensitivities around the imposition of sociocultural norms. Alignment among donors can facilitate government leadership, ensure that government priorities are addressed, and support strategic and intentional draw-down of donor investment.

The DPG-H donors in Nigeria established an SBC coordination committee within the DPG-H because the DPG-H drives the agenda of the Health Partners Coordination Committee. Placing the SBC committee within the DPG-H (and by extension under the purview of the Health Partners Coordination Committee, which is convened by Federal Ministry of Health) helps ensure that SBC priorities are both visible and aligned with the Government of Nigeria's health objectives.

### Recognize and Leverage the Differing Strengths of Private and Public Donors

Recognizing the contrasts between public and private donors is necessary to improve collaboration processes, transparency, and efficiency. Private foundations' ability to move money quickly and act nimbly can accelerate the progress of collaborations involving public funders, whose investment processes take longer (T. Wood, personal communication, October 22, 2018; R. Vezina, Harder + Co. personal communication, November 6, 2018). Similarly, private donors often have the flexibility to fund emerging or highly specialized research or programming, and as such can act as disrupters or catalysts within the broader development community. Conversely, public donors typically offer the broad investments, long-standing relationships with host-country governments, and staff on the ground that are critical for achieving sustained impacts at scale.

The Ouagadougou Partnership offers an example of this public-private collaboration. USAID, the William and Flora Hewlett Foundation, BMGF, and other private donors have worked closely together to highlight the need for increased attention to demand-side drivers of family planning use. The private donors have been instrumental in funding essential research and monitoring through mechanisms such as Track20 and national audience segmentation studies. USAID has supported ongoing advocacy, capacity strengthening, and regional SBC campaigns. Within host countries, it is important to mobilize domestic donors, including from the private sector. Business councils operate in many countries and bring together public- and private-sector donors.

### Demonstrate Commitment by Investing Resources: Time, Money, Networks, and Institutional Clout

Members of collaborations must have a vested interest in the collaborative activity, but they need not commit equal amounts of funding to participate as equals. Committing early and offering non-financial resources and influence are valuable too, but it is important to be transparent about how funding levels relate to decision-making roles (B. Schlachter, FP2020, personal communication, November 20, 2018; L. Dakan, David and Lucile Packard Foundation, personal communication, November 26, 2018). Similarly, it is critical that collaborations include not only technical experts but recognized leaders and those with the authority to make funding decisions. Ouagadougou Partnership's 2019 annual meeting provides an example of shared investment as a reflection of commitment. Although the Hewlett Foundation and BMGF were primary funders of the partnership and supported the annual meeting itself, USAID SBC implementing partners worked closely with the Ouagadougou Partnership's secretariat and major funders to advocate for attention to SBC as a theme of the meeting and organized several SBC-specific events at the meeting. This joint effort on the part of donors, partners, and the secretariat effectively elevated SBC within the meeting without undue burden on a single funder.

Members of collaborations must have a vested interest in the collaborative activity, but they need not commit equal amounts of funding to participate as equals.

### Use Honest Conversations About Failure to Inform a Joint Learning Agenda

Successful collaborations recognize failure as a chance to inform a learning strategy, rather than a reputational threat to a given funder or implementer.^16^ Many funders are reluctant to admit that their investments have not achieved intended goals, because they fear it will reflect poorly on their partners or their own management of the investment. However, philanthropic researchers and advisors emphasize that strong organizations use what they learn to improve.^16^ In aligning their investments, funders can celebrate what has worked for them individually and collectively but must also be prepared to acknowledge what has not. Having some agreed-upon goals as well as indicators of progress and success can help facilitate these honest conversations. Using an iterative learning approach, such as Responsive Feedback, that guards against both a failure of the theory of change and a failure of implementation may be helpful.^17^

In India, Bangladesh, and Rwanda, BMGF and the World Bank are working with local partners to use behavioral science to improve complementary feeding and dietary diversity for small children, maternal nutrition, and the performance of health care workers. The collaboration is intended to support innovations addressing challenges where ongoing efforts were not seeing sustained results. The grant includes a specific component for capacity building and knowledge sharing of partner programs. This approach allows the project strategy to be continually refined and also enables the World Bank team to identify areas of work and propose solutions focused, in some cases, on adapting and improving the existing strategies being implemented. This effort includes identifying the need to revisit growth tracking but from the point of view of parents' aspirations instead of the traditional focus on monitoring program performance.

### Encourage Proactive Communication and Informal Discussion

Promoting a sense of full collaboration outside of formal, scheduled meetings can encourage team members to communicate as issues arise and build trust with one another. Members of strong collaborations stress the importance of cultivating trust among new and existing members and establishing expectations and habits that facilitate constructive relationships over time. It is important to recognize that each individual in the group represents an organization and may need time to sensitize the organization and navigate the organization's priorities and ways of working (V. Gauri, World Bank, personal communication, November 20, 2018; R. Vezina, Harder + Co., personal communication, November 6, 2018).

At Design for Health, a partnership between BMGF and USAID to promote the application of design practices in global health, development of strong relationships between a core group of staff at the 2 donor organizations allowed the team to course correct as needed, particularly when the implementing partners' work began to lean toward the strategic priorities of a single funder.

### Take the Time to Understand Collaborating Organizations' Grantmaking, Procurement, and Compliance Processes

Members of a collaboration must recognize each participating organization's administrative requirements, which may vary widely. Underestimating the time and resources needed to navigate procedures for making a grant, including procurement, due diligence, contracting, and reporting, will cause delays. To avoid this, technical experts may wish to engage colleagues focused on management, procurement, and compliance early in the development of a collaborative activity so that timelines and expectations are realistic. Doing this can also allow donors to leverage each other's funding streams effectively and efficiently.

For example, Design for Health was able to use BMGF's flexible funding for priority activities, such as community-building convenings, that USAID could not fund. In turn, USAID funding supported the development of public goods that were created as a follow-on to the community-building convenings. At the outset, USAID and BMGF discussed how they could best leverage their respective funding to achieve the partnership's strategic objectives.

### Consider Using a Trusted Member or an Intermediary to Progress Work

An intermediary can oversee the collaborative effort,^6^ providing facilitation and leadership, which allows the donor organization staff to engage as funders rather than as process-facilitators (J. Rangel de Almeida, Wellcome Trust, personal communication, November 9, 2018; L. Sussman, USAID, personal communication, October 17, 2018; T. Wood, BMGF, personal communication, October 22, 2018). Forming a secretariat that can take work forward on behalf of the group can be a useful approach (V. Winder, FP2020, personal communication, November 21, 2018).

In the CI approach to collaboration, this function is called “backbone support.” For example, Alive & Thrive (A&T) has provided backbone support for a multilayered SBC CI initiative, funded by BMGF and Irish Aid in Southeast Asia. A&T organized large events with UNICEF to build and maintain momentum around infant and young child feeding policy enhancement in seven countries in southeast Asia. At the country level, A&T strategized with the actors and provided them with capacity-building opportunities to advance policy work. They were able to mobilize funding to complement the existing resources and ensure ownership from the government and other organizations.

### Early Wins Build Confidence in the Group

Early wins demonstrate the value of working together and are essential to holding a collaborative group together.^18^ Writing about the CI approach, consulting group FSG recommends that groups pursue a^14^:

*portfolio of strategies that offer a combination of easy but substantive short-term wins to sustain early momentum for the initiative, as well as more ambitious, long-term systemic strategies that may not show impact for several years.*

One example of an “early win” from the SBC Donor Group convened by BMGF, USAID, and CIFF resulted from participants' sharing information about their investments. In preparation for the group's initial meeting in December 2018, participants provided information to BMGF about their organization's investments in SBC in prioritized countries. BMGF and a consultant collated this information and reformatted it into a database and maps of donors' SBC investments in selected countries. Participating donors identified overlaps and gaps in their investments. The tangible output was useful in the donors' individual planning of their investments. The database enables group members to analyze the portfolio of investment across donors, brings visibility to innovative donor initiatives, and encourages donors to explore similar investments for collaboration. Since then, the World Bank eMBeD unit has assumed management responsibility for the database.

## CONCLUSIONS

Since 2018, there has been a marked increase in collaboration among funders of SBC in global health. Anecdotal evidence suggests that these efforts are beginning to yield results, including reduced duplication of effort, improved support to implementers, and increased co-investment.

In 2020, the SBC Donor Group met remotely twice and discussed how donors were responding to the coronavirus disease (COVID-19) pandemic in the area of risk communication. The group's first meeting of 2021 addressed expanding the use of SBC for health systems change rather than limiting its use to individual behavior change. The group intends to support agenda-setting for the next International SBCC Summit (scheduled for December 2022) and will likely use this forum to assess the group's results to date, expand membership with more donors based in countries of implementation, and identify specific opportunities for collaboration and cofunding.

Moving forward, funders must be intentional in building communities and collaborative relationships that both leverage these early successes and strive to apply proven practices such as those discussed in this article. It is critically important that funders institutionalize collaborations, moving beyond a small number of participating donors and their staff to engage a broader range of organizations and individuals with a vision and mission that resonates broadly and is supported by decision makers within each participating organization. Achieving this broader participation will require both documentation of the results of donor collaboration and targeted outreach, with attention to smaller funders and organizations that have not historically invested heavily in demand-side programming. As collaborations, such as the SBC donor group initiated by BMGF, USAID, and CIFF, mature, it will be important that they move beyond sharing information to enable more integrated coordination, co-investment, and even cocreation of investments. Global and regional collaborations among donors must also seek to engage host country governments and regional coordinating bodies as leaders in the work of SBC.

### Suggested Next Steps

The OECD conducted research in 2003 and 2018 on private philanthropy and collaboration networks among philanthropists and with ODA. To the authors' knowledge, this manuscript is the first that considers donor collaboration focused specifically on SBC. The sector would benefit from additional research, including updated research on the barriers and facilitators to donor collaboration in SBC, from the perspective not only of donors, but also from the perspectives of host-country governments, of implementing organizations, and of intended beneficiaries.

## Supplementary Material

21-00007-Harbour-Supplement.pdf

## Footnotes

* Many readers will be familiar with the term social and behavior change communication (SBCC), defined as the integrated use of a range of communication approaches—mass media; “new” and social media; community-level activities; and interpersonal communication (IPC)—to influence norms and behaviors pertaining to health. The authors use the broader term, SBC, which is understood to encompass both SBCC and non-communication-based approaches to behavior change. SBC includes any approaches, activities, or interventions that directly affect knowledge, attitudes, behavior, and social norms that influence health or development outcomes. Such interventions may be grounded in several different disciplines, including SBCC, marketing, advocacy, behavioral economics, or human-centered design.

† The terms “supply” and “demand” are used in development assistance for strengthening health sector services to describe the availability of health services (supply) and individuals' use of those services (demand). Supply-side interventions may focus on improving the technical quality and/or accessibility of a health service, including geographic, economic, and cultural access. Demand-side interventions may focus on making intended individuals aware of available services and motivating them to seek out and use these services.

## Notes

**Peer Reviewed**
