# Supplementary material for: How Donors Can Collaborate to Improve Reach, Quality, and Impact in Social and Behavior Change for Health
Source: Glob Health Sci Pract. 2021 Jun 30;9(2):246–53. doi: 10.9745/GHSP-D-21-00007 (PMC8324193; doi:10.9745/GHSP-D-21-00007)
Supplement: 21-00007-Harbour-Supplement.pdf [file 21-00007-Harbour-Supplement.pdf]

Supplement to: Harbour C, Hempstone H, Brasington A, Agha S. How donors can collaborate to improve reach, quality, and impact in social and behavior change for health. *Glob Health Sci Pract*. 2021;9(2). https://doi.org/10.9745/GHSP-D-21-00007

Supplemental Text – Examples of Donor Collaborations

The Supplement gives more information and links (if they exist) for the examples of donor collaboration in SBC that are mentioned in Table 1, which appears in the main text.

Global SBC Donor group

This is a collaboration of funders of social and behavior change (SBC) interventions supported by the Bill & Melinda Gates Foundation and USAID with the objective of sharing donors' demand-side priorities and improving donor coordination.

Scaling Up Nutrition

The SUN Movement Strategy and Roadmap (2016-2020) has helped illuminate the importance of nutrition as a universal agenda – and one which is integral to achieving the promise of the Sustainable Development Goals (SDGs). To realize the vision of a world without hunger and malnutrition, the SUN Movement Principles of Engagement guide actors as they work in a multi-sectoral and multi-stakeholder space to effectively working together to end malnutrition, in all its forms.

The Curve Community of Practice

Curve is a global community of practice and a Nigeria based community of practice, supported by the Bill & Melinda Gates Foundation, where practitioners learn how to rely on programmatic feedback for evidence-based informed decisions.

Nigeria SBC Donor Coordination Committee, and similar groups in other countries

The Donor Partners Group for Health in Nigeria established an SBC coordination committee to drive the agenda of the government's health partners and ensure that SBC priorities are both visible and aligned with the Government of Nigeria's objectives.

Global Partnership to End Violence Against Children

The Global Partnership to End Violence Against Children was launched in 2016 to end all forms of violence against children. It is comprised of over 500 partners, including governments, UN agencies, research institutions, international non-governmental organizations, foundations, civil society organizations and private sector groups.

Social Norms Learning Collaborative

The Learning Collaborative to Advance Normative Change is **a** global and regional CoP funded by the Bill & Melinda Gates Foundation, USAID, and the William and Flora Hewlett Foundation to increase the understanding and application of normative approaches in social behavior change interventions.

Adolescents 360

Adolescents 360 is a partnership between the Children's Investment Fund Foundation and the Bill & Melinda Gates Foundation that supports a girl-centered approach to

Supplement to: Harbour C, Hempstone H, Brasington A, Agha S. How donors can collaborate to improve reach, quality, and impact in social and behavior change for health. *Glob Health Sci Pract*. 2021;9(2). https://doi.org/10.9745/GHSP-D-21-00007

contraceptive programming. A360 is supporting health systems across Nigeria, Ethiopia, Kenya and Tanzania to include a girl-centered approach, led by girls and national governments.

GenU (Generation Unlimited)

Generation Unlimited (GenU**)** is a global multi-sector partnership of government, multilateral organizations, civil society, the private sector, and young people from around the world to meet the urgent need for expanded education, training and employment opportunities for youth ages 10 to 24.

United Nations Trust Fund to End Violence Against Women

Link: UN Trust Fund to End Violence Against Women

The UN Trust Fund is a global grant-making mechanism that is dedicated exclusively to addressing all forms of violence against women and girls. The Fund is managed by UN Women on behalf of the UN System. The funds raised support initiatives by grantees that have a tangible and sustainable impact.

UReport

UNICEF's UReport uses technology to provide lifesaving information and services, develop new approaches to engage young people in change, and identify and support emerging technologies that enable the youth to be future ready.

Conrad N. Hilton Foundation and Bernard van Leer Foundation

This is a collaboration to support INSEAD to design and deliver a course for policy makers and program leaders on the use of behavioral science in early childhood learning,

Alive & Thrive in ASEAN

The Alive & Thrive (A&T) regional initiative in Southeast Asia is rooted in advocacy, support and implementation learning for increasing the practice of breastfeeding. It documents the financial and social cost of not breastfeeding and strives to synchronize maternity protection and workplace breastfeeding programs.
